# Does Rebound Tonometry Probe Misalignment Modify Intraocular Pressure Measurements in Human Eyes?

**DOI:** 10.1155/2013/791084

**Published:** 2013-08-29

**Authors:** Ian G. Beasley, Deborah S. Laughton, Benjamin J. Coldrick, Thomas E. Drew, Marium Sallah, Leon N. Davies

**Affiliations:** ^1^Ophthalmic Research Group, Life and Health Sciences, Aston University, Birmingham B4 7ET, UK; ^2^Biomedical Engineering Research Group, Engineering and Applied Sciences, Aston University, Birmingham B4 7ET, UK

## Abstract

*Purpose*. To examine the influence of positional misalignments on intraocular pressure (IOP) measurement with a rebound tonometer. *Methods*. Using the iCare rebound tonometer, IOP readings were taken from the right eye of 36 healthy subjects at the central corneal apex (CC) and compared to IOP measures using the Goldmann applanation tonometer (GAT). Using a bespoke rig, iCare IOP readings were also taken 2 mm laterally from CC, both nasally and temporally, along with angular deviations of 5 and 10 degrees, both nasally and temporally to the visual axis. *Results*. Mean IOP ± SD, as measured by GAT, was 14.7 ± 2.5 mmHg *versus* iCare tonometer readings of 17.4 ± 3.6 mmHg at CC, representing an iCare IOP overestimation of 2.7 ± 2.8 mmHg (*P* < 0.001), which increased at higher average IOPs. IOP at CC using the iCare tonometer was not significantly different to values at lateral displacements. IOP was marginally underestimated with angular deviation of the probe but only reaching significance at 10 degrees nasally. *Conclusions*. As shown previously, the iCare tonometer overestimates IOP compared to GAT. However, IOP measurement in normal, healthy subjects using the iCare rebound tonometer appears insensitive to misalignments. An IOP underestimation of <1 mmHg with the probe deviated 10 degrees nasally reached statistical but not clinical significance levels.

## 1. Introduction

The iCare TA01i (Icare Finland Oy, Helsinki, Finland) rebound tonometer is a relatively recent addition to the portfolio of tonometers currently available to the ophthalmic practitioner for measuring intraocular pressure (IOP). Hitherto, studies have shown that the rebound tonometer performs adequately as a screening tool in comparison to the Goldmann applanation tonometer (GAT; Clement Clarke International, Harlow, UK) and other handheld tonometry devices [1–4].

In brief, the iCare TA01i rebound tonometer comprises a solenoid and housing, a magnetised probe, and other associated electronics. The probe is 40 mm long, 0.3 mm in diameter with a 1.7 mm diameter plastic end-tip [5]. The device employs a solenoid to fire the probe to travel towards the cornea at a velocity of approximately 0.2 m/s. Following the propulsion pulse, electronics switch to monitor the voltage induced in the solenoid coil by the movement of the magnetised probe, allowing the speed and direction of probe movement to be monitored. Signal processing electronics and microcontrollers then derive the probe deceleration time on corneal impact and convert this to a measure of IOP.

As the iCare probe has a small footprint, it is possible to measure IOP in a number of corneal positions; this is particularly useful in the presence of central corneal abnormalities. What remains equivocal, however, is the impact of probe-cornea misalignments on the resultant IOP reading. Previous studies have attempted to elucidate these putative erroneous errors; however, to date, these studies have generally neglected to examine the central 4 mm corneal zone within which most readings would normally be acquired, clinically. Instead, studies have measured IOP at positions 2 mm from the limbus [6–8]. Further, the exact methods adopted to adjust probe position in relation to the cornea are often an approximation using rudimentary techniques [9, 10] and they generally lack any real quantifiable level of precision and accuracy [11].

The aim of the present study, therefore, was to assess the impact of iCare TA01i probe-cornea misalignments on IOP using a bespoke alignment rig with a high level of positional accuracy and precision. Here, measurements were acquired with horizontal angular deviations at 5° and 10°, both nasally and temporally. Further, in contrast to previous research, lateral misalignments were assessed within the central 4 mm corneal zone. 

## 2. Methods

Data were collected from the right eyes of 36 healthy subjects (16 men and 20 women) aged from 17 to 49 years (mean ± SD 24.3 ± 7.6 years). Informed consent was obtained from each subject, and the research followed the tenets of the Declaration of Helsinki. The School of Life and Health Sciences Human Ethics Committee at Aston University, Birmingham, United Kingdom, approved all procedures.

**Figure 1:**
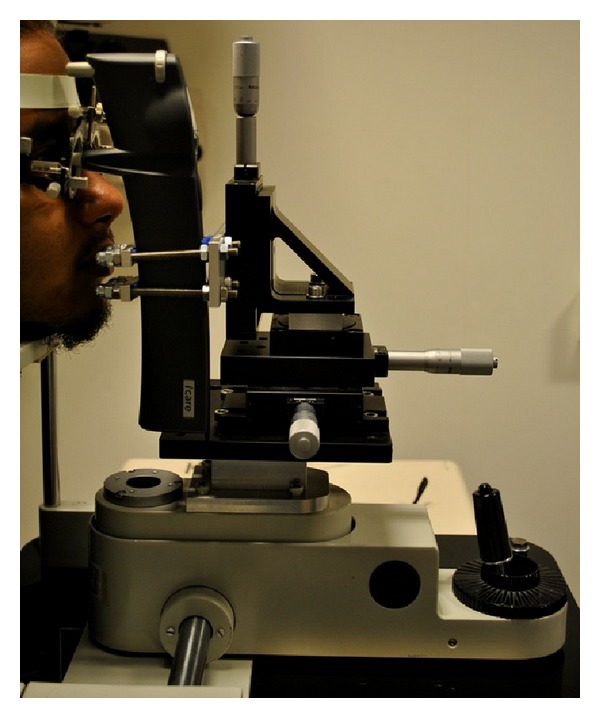
Side view of the subject with the headrest and slit lamp base to which the bespoke rig was attached and the iCare tonometer fixed in place. The micrometer scale gauges used to modify the tonometer's position can be observed.

IOP measurements were initially taken with the iCare tonometer. For the purpose of this study, the iCare was mounted onto a slit lamp base containing a bespoke alignment rig with six degrees of freedom, thus enabling precise and accurate manipulation of the probe position. The micrometer scales used to modify the tonometer's position in relation to the cornea afforded a 0.01 mm level of precision for linear displacements and 1° for angular deviations (see Figure 1). With the subjects seated, relaxed, and resting their heads on the slit lamp, baseline IOP measurements were acquired with the tip of the disposable iCare probe aligned normal to the corneal apex and 5 mm away from the anterior corneal pole; this distance was measured using the vertex distance scale mounted on an Oculus Universal Spring Cell Trial Frame (OCULUS Optikgeräte GmbH, Münchholzhäuser, Germany), which was worn for the entire duration of all iCare measurements to enable continuous monitoring of the eye-probe alignment.

To determine the effect of probe misalignments, IOP was measured with the iCare tonometer in multiple positions. Horizontal angular deviations included 5° nasally (5°N), 10° nasally (10°N), 5° temporally (5°T), and 10° temporally (10°T). In addition, the iCare probe was deviated laterally by ±2 mm nasally (2N) and temporally (2T). The sequence of data collection was randomised to control for the potential effect of measurement order. For all positions, three repeats of six consecutive readings were taken with the same iCare probe and averaged.

On completion of all iCare IOP readings, GAT measurements were taken. The GAT is currently the clinical gold standard for measuring IOP [12] and has been described in detail elsewhere [13]. Disposable Tonosafe probes were used for all GAT readings, a protocol adopted in previous investigations [5, 14], as repeated use of the original probe would otherwise require time-consuming sterilization procedures, unsuitable for the present study. Following a slit lamp examination of the anterior corneal surface, one drop of 0.5% proxymetacaine and 0.25% fluorescein (Minims, Bausch & Lomb Pharmaceuticals, Inc., FL, USA) was instilled into the right eye of each subject. 

A second UK-registered optometrist, who was blind to the tonometer scale and to the iCare baseline readings, took GAT measures. All tonometry readings were acquired within a 20-minute interval thus reducing the effect of diurnal changes [15]. Again, a thorough slit lamp examination was conducted following the procedure.

### 2.1. Statistical Analysis

All statistical analysis was carried out using SigmaPlot (version 12; Systat Software UK Ltd., London, UK) and SPSS for Windows (version 15; SPSS Inc., Chicago, IL, USA). All data were assessed to confirm normality (using the Kolmogorov-Smirnov test). A probability of <0.05 was taken as statistically significant. Differences in IOP measurements with the two instruments were calculated by subtracting the values obtained with the Goldmann tonometer from those obtained with the iCare tonometer. In addition, variations in iCare IOP values with probe deviation in angle and position were calculated. Paired two-tailed *t*-tests were used to determine any bias between the methods [16]. Limits of agreement between measurements made with GAT and iCare tonometer were expressed at the 95% level (mean of the difference ± 1.96 SD of the differences) and were also calculated as recommended by Bland and Altman [17, 18].

## 3. Results

### 3.1. iCare Measurements at CC versus GAT

**Figure 2:**
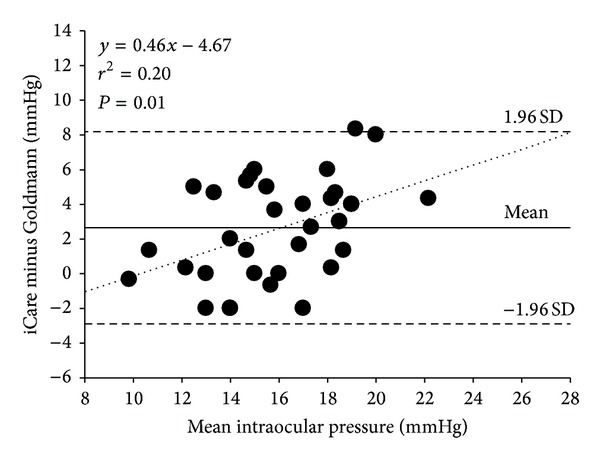
Difference *versus* the mean plot illustrating the comparison between IOP measurements taken with GAT and iCare tonometer. The iCare tonometer significantly overestimated IOP compared to the GAT. The solid line represents the mean bias, and the dashed lines represent the 95% limits of agreement.

The mean IOP ± SD, as measured by GAT, was 14.7 ± 2.5 mmHg *versus* central iCare tonometer readings of 17.4 ± 3.6 mmHg and represents a significant positive correlation (*r* = 0.446; *P* < 0.001) with an overestimate of 2.7 ± 2.8 mmHg (paired *t*-test: *t* = 5.628, *P* < 0.001) the bias of which increased at higher IOPs (see Figure 2). 

### 3.2. iCare Measurements at CC versus Specified Lateral and Angular Deviations

**Figure 3:**
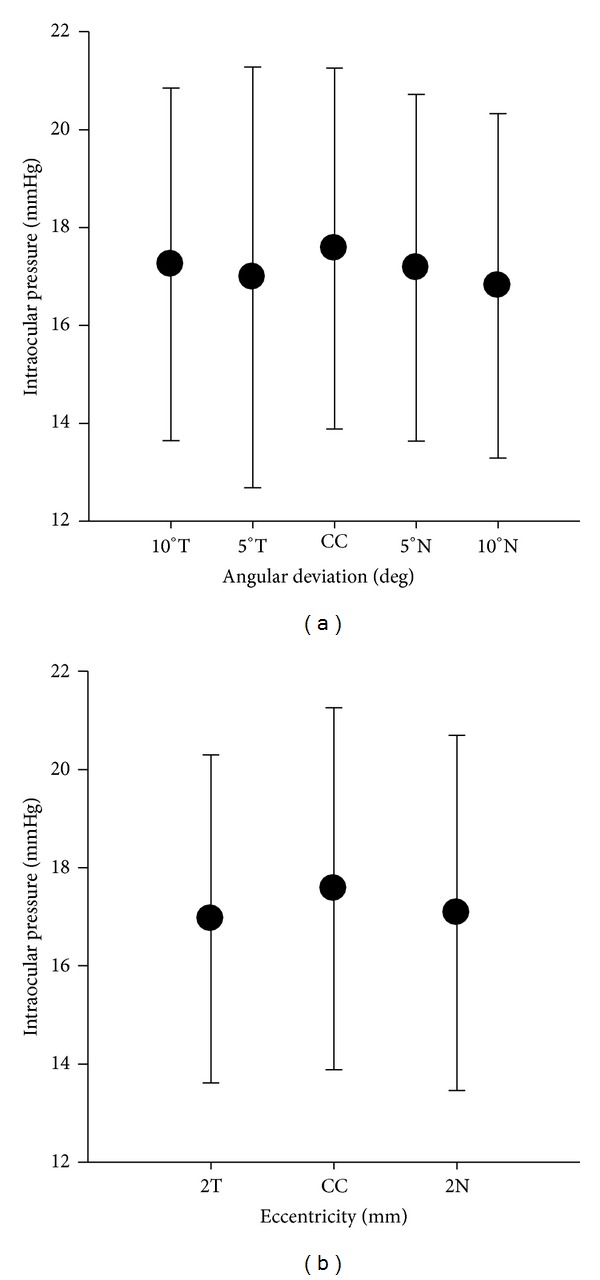
Mean iCare IOP measurements at CC *versus* specified lateral and angular deviations. Deviating the ICare tonometer probe from the optimal position had little effect on measurements, except at 10°N, where the underestimate of IOP reached levels of significance. Error bars represent ±1 SD.

Measures of IOP at CC using the iCare tonometer were not significantly different to those with lateral displacement of the probe at 2N (paired *t*-test: *t*= 1.18, *P* = 0.246) or at 2T (paired *t*-test: *t* = 1.295, *P* = 0.204). Comparisons were also made between the iCare IOP measurements taken at CC and angular probe deviations; these measures were statistically similar at 5°T (paired *t*-test: *t* = 1.229, *P* = 0.227), 10°T (paired *t*-test: *t* = 0.698, *P* = 0.491), and 5°N (paired *t*-test: *t* = 1.280, *P* = 0.209), but not at 10°N (paired *t*-test: *t* = 2.243, *P* = 0.031), which showed an underestimation of IOP compared with central corneal apex measures of 0.8 ± 2.1 mmHg (see Figure 3).

**Figure 4:**
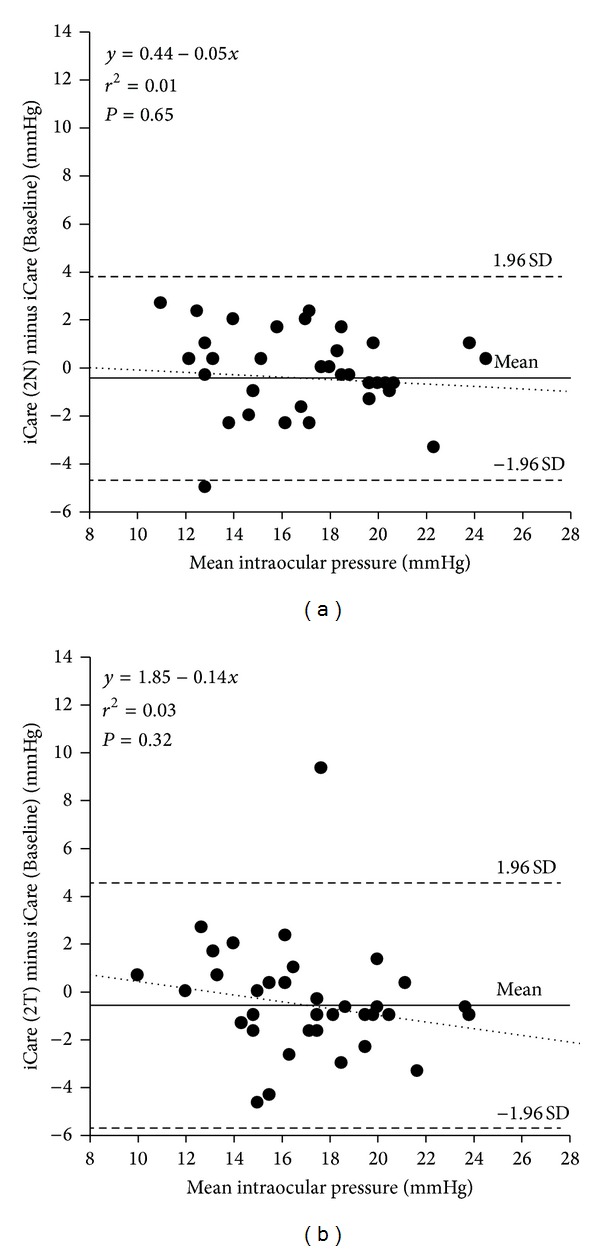
Difference versus mean plot illustrating the comparison between IOP measurements taken with the iCare tonometer at the baseline position (CC) and at specified lateral deviations. (a) iCare tonometer probe was laterally displaced 2 mm nasally from the centre of the cornea. (b) iCare tonometer probe was laterally displaced 2 mm temporally from the centre of the cornea. The solid line represents the mean bias, and the dashed lines represent the 95% limits of agreement with the dotted regression line displayed. Lateral deviation of the probe showed no significant correlation with measures at CC in nasal or temporal positions.

**Figure 5:**
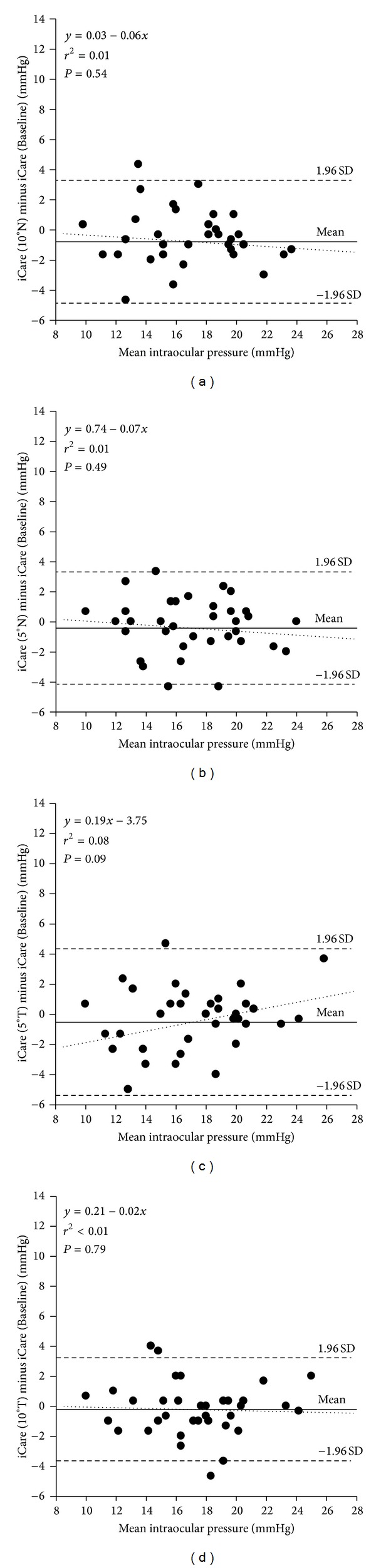
Difference versus mean plot illustrating the comparison between IOP measurements taken with the iCare tonometer at the baseline position (CC) and at specified angular deviations. (a) iCare tonometer probe was displaced by an angle of 10 degrees nasally. (b) iCare tonometer probe was displaced by an angle of 5 degrees nasally. (c) iCare tonometer probe was displaced by an angle of 5 degrees temporally. (d) iCare tonometer probe was displaced by an angle of 10 degrees temporally. The solid line represents the mean bias, and the dashed lines represent the 95% limits of agreement with the dotted regression line displayed. Angular deviation of the probe showed no significant correlation with measures at CC, in either of the two nasal and temporal positions.

Analysis showed no significant correlation between iCare tonometer measures of IOP at CC and those taken at a lateral deviation of 2 mm nasally (*r* = 0.079, *P* = 0.646) nor when measured 2 mm temporally (*r* = 0.171, *P* = 0.319) (see Figure 4). For angular deviations, analysis showed no significant correlation between iCare tonometer measures of IOP at CC and those taken at 5°T (*r* = 0.289, *P* = 0.088), 10°T (*r* = 0.047, *P* = 0.785), 5N (*r* = 0.118, *P* = 0.493), and 10°N (*r* = 0.103, *P* = 0.549) (see Figure 5).

## 4. Discussion

The purpose of the present study was to determine the effect of specified lateral and angular deviations of the iCare tonometer probe on the accuracy of IOP measurement as compared to readings in the optimal position, namely, the central corneal apex; this is the first time that a study of this type has used a bespoke rig to carefully control the desired iCare tonometer probe positions. Further, the relatively small lateral and angular deviations specified in the present study, confined to the central 4 mm zone, were chosen to assimilate the typical misalignments that could occur in everyday clinical practice. IOP measurements taken at the central corneal apex were also compared to the current clinical gold standard, the Goldmann applanation tonometer, in normal, healthy subjects.

The present study has confirmed, in broad agreement with earlier work [11, 19, 20], that in comparison to GAT, the iCare tonometer overestimates IOP, with this bias widening at higher readings. That is to say, the iCare overestimates IOP for patients with higher IOP values.

For lateral deviations, the iCare tonometer readings were marginally lower than those at the central corneal apex (see Figure 3) but not at levels of statistical significance either at nasal or temporal eccentricities. Similarly, for angular deviations of 5 and 10 degrees temporally, and 5 degrees nasally, iCare tonometer readings were lower than readings with the instrument in its optimal position, but not reaching levels of statistical significance. However, when the iCare tonometer probe was deviated 10 degrees nasally, the underestimate of IOP did reach a significant level statistically, although this <1 mmHg difference is of little consequence in a clinical context considering the intrasubject variability of Goldmann-type tonometers [21].

A principal advantage of the iCare rebound tonometer is its ability to measure IOP in patients who are supine at the time of examination. Although our device was able to control accurately the position of the upright iCare tonometer, our study did not examine the influence of patient position on the resultant IOP value. A further study, therefore, is indicated to examine the influence of probe position (upright *versus* horizontal) on IOP readings in healthy and diseased eyes. In this regard, it would also be interesting to examine patients with corneal pathology (e.g., band keratopathy), which may, in turn, influence the resultant IOP value measured with the iCare.

## 5. Conclusions

The present study has shown that the iCare tonometer is clinically robust to small but tangible lateral and angular deviations that can occur during the use of a handheld instrument in general ophthalmic practice.
